# Understand the Changes in Motivation at Work: Empirical Studies Using Self-Determination Theory-Based Interventions

**DOI:** 10.3390/bs15070864

**Published:** 2025-06-25

**Authors:** Zheni Wang, Melanie Briand

**Affiliations:** 1 School of Business, Southern Connecticut State University, New Haven, CT 06515, USA; 2 John Molson School of Business, Concordia University, Montreal, QC H3G 1M8, Canada; melanie.briand@concordia.ca

**Keywords:** motivation change, employee training and development, self-determination theory, theory-based interventions

## Abstract

Managers often need to stay motivated and effectively motivate others. Therefore, they should rely on evidence-based interventions to effectively motivate and self-motivate. This research investigated how self-determination theory-based interventions affect employees’ motivation dynamics and motivational consequences within short time frames (i.e., within an hour, within a few weeks or months) in two empirical studies. Study one focused on assessing the effectiveness of a one-day training workshop in helping to improve managers’ work motivation, basic psychological needs satisfaction/frustration, subordinates’ motivation, and perceptions of managers’ needs-supportive/thwarting behaviors within a few weeks. Results support the effectiveness of the training, as managers were rated by their direct subordinates as having fewer needs-thwarting behaviors and reported self-improvement in needs satisfaction and frustration six weeks after completing the training program. Study two used the mean and covariance structure analysis and tested the impact of three types of basic psychological needs-supportive/thwarting and control conditions (3 × 2 × 1 factorial design) on participants’ situational motivation, vitality, and general self-efficacy for playing online word games within 30 min. Multi-group confirmatory factor analysis (CFA) confirmed the scalar measurement invariance, then latent group mean comparison results show consistently lower controlled motivation across the experimental conditions. During a quick online working scenario, the theory-based momentary intervention effectively changed situational extrinsic self-regulation in participants. Supplementary structural equation modeling (SEM; cross-sectional) analyses using experience samples supported the indirect dual-path model from basic needs satisfaction to vitality and general efficacy via situational motivation. We discussed the theoretical implications of the temporal properties of work motivation, the practical implications for employee training, and the limitations.

## 1. Theoretical Framework

**Figure 1:**
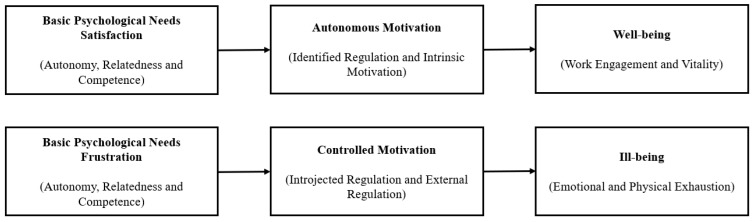
Dual-path model from needs satisfaction/frustration to psychological health via motivation (adapted from B. Chen et al., 2015).

Self-determination theory (SDT; Deci & Ryan, 2000; Ryan & Deci, 2017; Ryan, 2023), a classic motivation theory widely applied to modern management practices (Ryan, 2023), differentiates not only among levels (i.e., quantity) of motivation, but also among the dimensions (i.e., quality) of motivation. This motivation theory proposes that people engage in activities as a result of different types of intrinsic or extrinsic self-regulation (Ryan & Deci, 2000, 2017). The SDT dimensional motivation model (Deci & Ryan, 2000) suggests that people can be motivated both by controlled drives (e.g., to obtain monetary compensation, to avoid punishment, and/or to avoid guilty feelings) as well as autonomous drives (e.g., to have fun performing a given task or to realize one’s values or beliefs). Autonomous and controlled motivation are not opposites; each encompasses different types of psychological drive and motives that primarily result from distinct regulatory styles, and each predicts distinct cognitive and behavioral outcomes (Gagné & Deci, 2005; Ryan & Deci, 2017). SDT (Deci & Ryan, 2000, p. 229) proposes basic psychological needs as “innate psychological nutriments that are essential for ongoing psychological growth, integrity, and well-being.” Hence, people are naturally inclined to seek out needs-satisfying activities, both implicitly and explicitly, to grow, connect, master challenges, and integrate new experiences. However, these natural tendencies do not operate automatically, but require constant and consistent social “nutriment” and support (Deci & Ryan, 2000, 2008). The three basic psychological needs are the needs for autonomy (Chirkov et al., 2003), relatedness (Baumeister & Leary, 1995), and competence (Csikszentmihalyi, 1988), which are essential to human psychological health and, ultimately, to human functioning (Sheldon & Filak, 2008). SDT posits that both autonomy, expressed through volitional and holistic self-regulation, and harmony, achieved through the integration of oneself with others, are essential to human flourishing (Ryan & Deci, 2017; Ryan, 2023). Both tendencies serve as fundamental aspects of human life that cannot be taken for granted (Deci & Ryan, 2008). The social environment can either facilitate and enable or disrupt and fragment human developmental processes, with the former leading to human flourishing and the latter resulting in significantly less desirable consequences (Deci & Ryan, 2008). Hence, the psychological function of human beings should reflect their ability to achieve growth (i.e., well-being) through the satisfaction of needs and successful social interactions across various life domains. It should also reflect their ability to avoid sickness and exhaustion (i.e., ill-being; see Figure 1 for the dual-path model) that results from not integrating with their social environment due to a lifetime of needs frustration/interpersonal thwarting (Ryan & Deci, 2017; B. Chen et al., 2015).

The present research focused on investigating and utilizing changes in motivation as a theory-based manipulation (i.e., training or experimental interventions) in needs satisfaction/frustration forming a critical predictor of changes in human attitudes and behaviors, which are often described as human learning behavior (De Houwer et al., 2013) in many different organizational settings. Theory-based intervention research is critically needed to advance our understanding of how to foster sustainable work motivation and well-being. Without a clear theoretical basis, interventions risk being fragmented or ineffective, limiting their contribution to both scientific knowledge and practical application (Deci et al., 2017). Moreover, theory-based designs ensure that interventions are systematic, replicable, and adaptable across organizational contexts (Ryan, 2023). Embedding intervention research within the SDT framework not only helps refine the theory itself but also provides actionable insights for building more motivating and human-centered workplaces (Gagné & Deci, 2005; Ryan, 2023).

Evidence from this research can inform the design and implementation of effective managerial development and organizational change programs (Hastings & Schwarz, 2022), guided by empirical evidence informed by self-determination theory (SDT). Organizations need effective theory-based interventions to support effective employee development programs. Managers often play a crucial role in motivating and supporting employees in their daily work. Managers’ motivational style, defined by Hardré and Reeve (2009, p. 167) as “the way a manager seeks to motivate employees in the workplace,” indicates a range of their behavior for promoting a high quality of motivation in their subordinates effectively. These types of motivating behavior could be described as “needs-supportive,” which includes, but is not limited to, acknowledging the perspective of their employees; providing employees with information and meaningful rationales; offering choice; encouraging self-initiation; giving effective feedback (change-orientated vs. promotion-oriented; Carpentier & Mageau, 2013); setting realistic goals; and showing empathy, concern, and appreciation (Su & Reeve, 2011). At the same time, some organizational practices may not only fail to support people’s basic psychological needs, but also actively thwart these essential psychological nutrients that facilitate effective self-regulation (B. Chen et al., 2015). For example, depletion of support and resources to deliver high-demanding performance under tight deadlines, abusive supervision, social segregation, and learned helplessness; these phenomena that lead to needs-frustration often positively predict employee ill-being (Ryan & Deci, 2000). Hence, assuming managers could adapt to the differences in their subordinates and situations/tasks as the context requires, their ability to carry out the actual attitude, for example, the values, beliefs, motivation to lead, and behavior, becomes the key to their effectiveness in motivating themselves and others at work. These assumptions differ significantly between the intervention conditions evaluated in this research and those of other typical leadership intervention studies. We aim to systematically develop and integrate a set of actionable, evidence-based, and theory-driven motivation interventions into managers’ daily toolboxes, promoting individual and organizational advancement.

This empirical research yields several benefits. First, we aim to validate the theory that interventions based on theory can be used to intentionally initiate changes in motivation in both managers and their subordinates. Our primary research hypothesis is that theory-based interventions can trigger a change in motivation and motivational consequences at work through purposeful alterations (i.e., providing needs-supportive feedback in study one, supporting or thwarting basic psychological needs in study two) in the basic psychological needs within daily working events. Second, we hope to advance the SDT framework to accommodate the dynamics of different types of short-term (i.e., hours, days, and months) motivational changes and their cognitive, attitude, and behavioral consequences across a variety of conceptual levels (Vallerand, 1997). Finally, we adopt both field and online experiment research designs in this research to imply and maximize the causal mechanisms underlying changes in motivation at work.

This research, comprising two independent studies, investigated how to change employees’ work motivation and needs-supportive/thwarting behavior by increasing satisfaction and minimizing the frustration of employees’ basic psychological needs for autonomy, relatedness, and competence within the work context. With both studies one and two focusing on understanding the short-term change in motivation toward organizational settings (i.e., instructions given by managers or a systematic management training program), we conducted two independent experiments. The first study was a field study that assessed the effectiveness of a one-day training intervention, aiming to improve managers’ autonomous motivation and change employees’ perceptions of their managers’ needs-supportive and thwarting behaviors. The second online study employed a 3 × 2 × 1 factorial design to investigate the effects of momentary needs-supportive and thwarting instructions on situational motivation, vitality, and general efficacy when participants were instructed to play online cognitive word games for a short period.

**Study One**

A one-day training workshop using the SDT framework was planned, which includes multiple training modules explaining the conceptualization of motivation and basic psychological needs, as well as experiential activities to observe, assess, and act supportively across different work situations. This field study was conducted in a small Canadian government agent upon a training invitation. Due to the small size of the managerial training sections and schedule limitations, no control groups were established. Instead, using a pre- and post-experimental design, we repeatedly measured and analyzed key variables before and after the training section for this field study.

## 2. Study One—Methods

### 2.1. Study One—Participants

Participants were 22 managers (*n* = 22; 75% male; 80% with bachelor’s degrees and up; mean age = 47 yrs., and SD = 6.4 yrs.) in a small government agency. In addition, two groups of 11 managers were provided with a one-day workshop at different times.

### 2.2. Study One—Procedures

The one-day training workshop includes modules and experiential learning activities to develop needs-supportive management competence in managers with supervisory responsibilities, such as how to properly speak and listen to employees, how to stimulate employee cooperation, how to promote employee involvement, how to make your employees feel valued, and how to encourage employees to modify behavior using proper feedback. The design of the training workshop utilized past research evidence on different types of needs-supportive feedback and its effectiveness in enhancing well-being and performance in sports psychology (Lemelin et al., 2022). The underlying assumptions of this training workshop lie in raising the managers’ self-awareness by changing his/her feedback-providing styles to become more needs-supportive and avoid being needs-thwarting toward their subordinates in controlling situations.

Repeated measures (before training–T1 and after training–T2) were obtained both from participating managers and their subordinates at two different times (T1 and T2); one was two weeks before the training workshop date, and the other was six weeks after the training workshop. A total of 87 subordinates (81.6% male; mean age = 39 yrs., and SD = 10 yrs.) were invited to fill out surveys about their direct managers before (T1) and after (T2) the training workshop, and a total of 61 employees (final *n*’ = 61) were matched to have filled both the before and after training survey questionnaires.

### 2.3. Study One—Measures

#### 2.3.1. Motivation at Work

The motivation at work scale (MAWS; Gagné et al., 2015) was used by both managers (T1 *Cronbach’s α* = 0.70; T2 *Cronbach’s α* = 0.71) and employees (T1 *Cronbach’s α* = 0.75; T2 *Cronbach’s α* = 0.76). Participants were asked to use a Likert scale from 1 to 7 (1 corresponds to not at all for this reason and seven corresponds to totally for this reason) to evaluate the statement to answer the question “why are you putting efforts in your work.” The MAWS has 19 items in total. Three items capture intrinsic motivation (i.e., “Because the work I do is interesting”), and three items capture identified regulation (i.e., “Because putting efforts in this job aligns with my personal values”). Six items measured extrinsic regulation (i.e., “To avoid being criticized by others such as supervisor, colleagues, family, clients.”), three items measured introjected regulation (i.e., “Because otherwise, I will feel bad about myself.”), and four items measured amotivation (i.e., “I don’t know why I’m doing this job, it’s pointless to work.”). We summed the intrinsic motivation and identified the regulations to count for autonomous motivation. At the same time, the extrinsic and introjected regulations for controlled motivation were accounted for accordingly (Ryan & Deci, 2017).

#### 2.3.2. Basic Psychological Needs Satisfaction/Frustration

The work-related basic psychological needs satisfaction scale (WBPNS; Van den Broeck et al., 2010) was used by managers (T1 *Cronbach’s α* = 0.82; T2 *Cronbach’s α* = 0.81) and employees (T1 *Cronbach’s α* = 0.85; T2 *Cronbach’s α* = 0.89) repeatedly to self-report the satisfaction of the basic psychological needs for autonomy, relatedness, and competence. Participants were asked to use a Likert scale from 1 to 7 (1 corresponds to strongly disagree and 7 corresponds to strongly agree) to evaluate workplace statements. The WBPNS has a total of 12 items. There are four items for each subscale, capturing satisfaction of basic psychological needs for autonomy (i.e., “I feel making choices about the way I work”), relatedness (e.g., “I feel part of a group”), and competence (e.g., “I feel competent at my job”).

The basic psychological needs frustration was measured using the scale developed by Bartholomew and colleagues (Bartholomew et al., 2011). Both managers (T1 *Cronbach’s α* = 0.77; T2 *Cronbach’s α* = 0.73) and employees (T1 *Cronbach’s α* = 0.85; T2 *Cronbach’s α* = 0.86) repeatedly reported their needs frustration at work using a Likert scale from 1 to 7 (1 corresponds to strongly disagree, while 7 corresponds to strongly agree) to assess statements about work. This scale has a total of 18 items. There are six items for each subscale, capturing frustration of basic psychological needs for autonomy (i.e., “I feel under pressure to agree with the workload I am provided.”), relatedness (i.e., “… I do not really mix with other people.”), and competence (i.e., “… I doubt whether I am able to execute my job properly”).

#### 2.3.3. Perceived Needs Support/Thwart Behavior

The perceived needs support and thwarting scale (Moreau & Mageau, 2012) was used for managers (T1 *Cronbach’s α* = 0.82; T2 *Cronbach’s α* = 0.81) to self-assess and for employees (T1 *Cronbach’s α* = 0.82; T2 *Cronbach’s α* = 0.81) to report their direct supervisor’s (i.e., manager) needs-supportive and thwarting behavior. Participants were provided with a Likert scale from 1 to 7 (1 corresponds to strongly disagree, while 7 corresponds to strongly agree) to assess statements describing managerial behaviors for themselves or direct supervisors. The perceived needs support and thwarting scale has a total of 21 items. A total of nine items capture needs-supportive behavior such as offering choice, explaining the reasons behind the demands and rules, and being aware of, accepting, and recognizing others’ feelings. Sample items for perceived needs-supportive behavior are “my supervisor gives me many opportunities to make decisions in my work,” and “my supervisor takes the time to listen to my opinion and point of view when I disagree with him/her.” There are 12 items capturing needs-thwarting behavior, such as inducing guilt, using threats, manipulating others by offering rewards, and giving orders. Sample items for perceived needs-thwarting behavior are “at times, my supervisor threatens to take away various privileges in order to pressure me into doing things differently,” and “my supervisor does not take the time to ask me to do something; he/she orders me to do it.”

### 2.4. Study One—Training Effectiveness Assessment Strategies

Profound learning effects from experiential learning may occur at different times in various situations. Therefore, we followed the traditional Kirkpatrick’s training program evaluation model (Kirkpatrick & Kirkpatrick, 2006) and assessed the immediate satisfaction of the training program via short questionnaires collected from the managers who completed the training on-site. As a result, overall positive ratings (more than 90% of the participating managers) regarding the training content, instructor, and delivery method were received right after the workshops were delivered. On the other hand, field training research involving managers and their subordinates ensures the relevance and validity of the potential research results. Both subordinate ratings and managers’ self-reports on their needs satisfaction/frustration and motivation help to cross-reference and increase the external validity of research findings.

To capture and assess the higher level of effectiveness of the training program, such as attitude improvement and behavior changes, we then conducted a series of mean comparative statistical analyses on repeated measurements (T1 vs. T2) to see how the learned concepts and skills could transform managerial behavior at work six weeks after the training workshop took place.

## 3. Study One—Results

Table 1 contains means and standard deviations for all the self-report variables and the correlations among these variables for managers and their direct subordinates for both T1 and T2.

Then, a series of univariate repeated ANOVAs (see Table 2) were conducted to examine changes in (1) the managers’ self-reported work motivation, basic psychological needs satisfaction/frustration, and needs-supportive/thwarting behavior; (2) employees’ assessment of their work motivation, needs satisfaction/frustration, and managers’ needs-supportive/thwarting behavior changed. Compared to baseline measures, managers self-reported a marginally lower level of need-thwarting behavior (F [1,43] = 3.2, *p* = 0.09, and ŋ^2^ = 0.11); and a significantly lower level of needs frustration (F [1,43] = 23.5, *p* = 0.00, and ŋ^2^ = 0.48); as well as a significantly higher level of needs satisfaction (F [1,43] = 7.46, *p* = 0.01, and ŋ^2^ = 0.23) after the training intervention. At the same time, their direct subordinates also reported a significantly higher level of needs satisfaction (F [1,121] = 28.2, *p* = 0.00, and ŋ^2^ = 0.19) and a lower level of needs frustration (F [1,121] = 5.05, *p* = 0.03, and ŋ^2^ = 0.09), as well as their manager demonstrating less needs-thwarting behavior (F [1,121] = 5.08, *p* = 0.03, and ŋ^2^ = 0.09) six weeks after supervisors went through the training intervention. The size of this training effect was around a small to moderate level.

Study Two

The hierarchical model of self-determined motivation (“H-SDT”; Vallerand, 1997) illustrates that self-determined motivation can act at three reciprocally related conceptual levels: the global (i.e., motivational traits such as trait), the life domain level (i.e., motivation for work vs. leisure), and the situational (i.e., while performing a specific activity or task). The H-SDT model supplements the basic tenets of SDT (Ryan & Deci, 2017), which primarily focuses on general and domain motivation, suggesting that situational motivation may fluctuate as people (e.g., employees) engage in different activities across various life domains at different times, such as work and leisure contexts. At the same time, this study also intended to expand the empirical investigation of motivation change at the situational level within a short temporal span (i.e., less than one hour).

**Figure 2:**
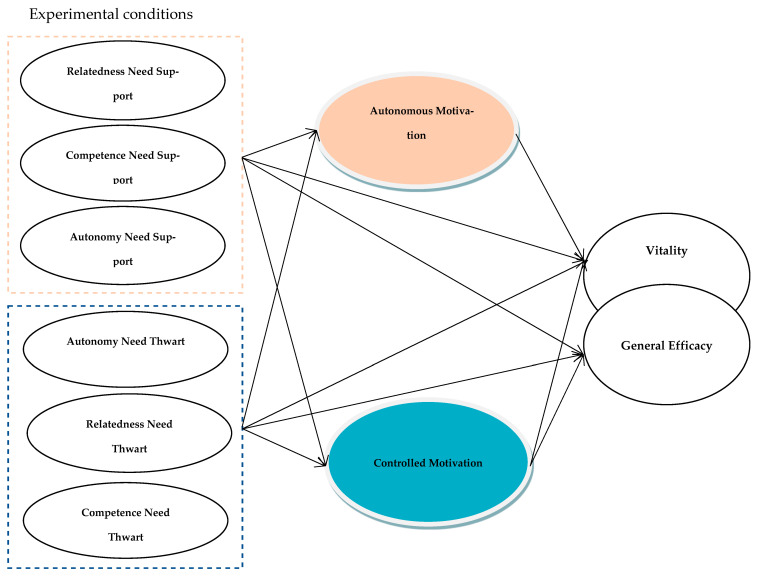
Research model of 3 × 2 × 1 factorial design online study two.^1^

Accumulated research investigating the perceived satisfaction and thwarting of basic psychological needs by significant others (e.g., life partners, coaches, teachers, and supervisors) suggests that satisfaction or frustration of these needs leads to well-being or ill-being, respectively (Deci & Ryan, 2008). While evidence shows that people’s well-being levels decrease with lower levels of needs satisfaction, it is also likely that active disturbances, such as specific actions/interactions that thwart psychological needs, are detrimental to people’s growth and adaptation in different contexts (Vansteenkiste & Ryan, 2013; Sheldon & Filak, 2008). For example, Gillet and colleagues (Gillet et al., 2012) found that perceived organizational and personal support impacted employees’ well-being and ill-being through their satisfaction and frustration with basic psychological needs resulting from work tasks via two distinct paths (see Figure 2 for the dual-path model). Moreover, empirical evidence supported the satisfaction of all three basic psychological needs—autonomy, relatedness, and competence—as essential factors for people’s momentary and developmental thriving across multiple life domains (i.e., education and sports; Sheldon & Filak, 2008). However, limited experimental studies examine the changes in momentary needs satisfaction and frustration caused by experimental manipulations of situational work motivation and employees’ well-being simultaneously (Ryan, 2023).

This experimental study synthesizes the H-SDT (Vallerand, 1997) and the dual-path framework (B. Chen et al., 2015; Ryan & Deci, 2017) under the SDT framework, specifically looking at the dual-path changes in situational motivation and employees’ subjective vitality and perceived general efficacy during a relatively short temporal duration, such as 20–30 min, in online settings. The experimental manipulations encompass all three basic psychological needs (autonomy, competence, and relatedness) through two independent paths (support vs. thwart) in a factorial design (see Figure 2 for the research model of the online study).

## 4. Study Two—Methods

### 4.1. Study Two—Participants

Participants were 374 (162 in three needs support conditions; 161 in three needs-thwarting conditions; and 49 in control conditions; see Section 2.3) American full-time corporate employees (57% male; 77% with bachelor’s degree and up; mean age = 36.4 yrs., and SD = 11.03 yrs.) recruited from Amazon MTurk with a small paid fee (USD 1 for approximately 20–30 min working on word games as well as filling out short survey questionnaires).

### 4.2. Study Two—Procedures

After obtaining informed consent, participants were asked to complete the online demographic questionnaire, which included questions about their age, education, and employer background. In addition, a baseline self-report on their situational motivation and general self-efficacy was also obtained (T1). Participants were then randomly assigned to one of the six experimental and control conditions to play two rounds of simple online word games; the first was to search for words related to workplace safety, and the second was to search for words related to healthy eating (see Appendix A for illustrations on experimental and control conditions and word game tasks). Each word game allowed participants three minutes of performance time, with a clock timer displayed on the online survey web page. In the end, participants were also asked to report on their situational motivation and subjective well/ill-being once again (T2) after they accomplished their word game activities.

### 4.3. Study Two—Experimental Manipulations

Participants were given general instructions about the online word search games: “You are chosen to do a few quick cognitive exercises to be selected for the next managerial training program (the required employees’ developmental program for the candidates to be promoted to supervising/managerial positions) in your organization. It is a simple task that requires both accuracy and efficiency. Therefore, please read the instructions for the exercise carefully.” The supportive conditions of basic psychological needs of autonomy, competence, and relatedness were based on the fundamental conceptualizations and typical procedures of past SDT research (Deci et al., 1994; Reeve et al., 2004; and Sheldon & Filak, 2008); at the same time, the thwarting conditions of basic psychological needs of autonomy, competence, and relatedness were also edited based on conceptualization and validated psychometric measurements (Sheldon & Filak, 2008; B. Chen et al., 2015; Ryan & Deci, 2017). For the detailed list of each experimental condition, please see the Appendix A. With 49 participants in the control condition, there were 162 participants included in the three needs-supportive (50 in autonomy, 58 in relatedness, and 54 in competence need-supportive) conditions and 163 participants in the three needs-thwarting (49 in autonomy, 62 in relatedness, and 52 in competence needs-thwarting) conditions.

Similar to many other game activities in past SDT experimental studies, online word games are simple, intrinsically interesting, yet challenging cognitive activities. Therefore, these short online activities are commonly valid and provide a reasonable work scenario to assess the interactions between individual and contextual factors for dynamics in human motivation. The Qualtrics survey platform captured each participant’s time spent and clicks on the word games for answers. The average time spent on each word game was 210.9 s (SD = 107.56 s), while the average number of clicks on each game was 7.7 (SD = 8.5) times. Although the average time spent on each word game was longer than the performance timing, which was 3 min (180 s), these statistics supported the participants’ strong situational motivation to work, which could be either autonomous or controlled, to complete the tasks as instructed in the study.

### 4.4. Study Two—Measures

#### 4.4.1. Situational Motivation

The situational motivation scale (Guay et al., 2000; 16 items, *Cronbach’s α* = 0.88 combined T1 and T2) was used to capture the situational motivation of the participant before and after the experimental conditions. In the situational motivation scale, participants were asked to answer the question “Why were you engaged in this activity” using a scale of 1 to 7 (1 corresponding to “not at all” and 7 corresponding to “exactly”). The scale has 16 items in total. Fousr items capture intrinsic motivation (i.e., “because I think that this activity is interesting”; *Cronbach’s α* = 0.93 for T1; *Cronbach’s α* = 0.95 for T2; and *Cronbach’s α* = 0.91 combined T1 and T2), four items capture identified regulation (i.e., “because I am doing it for my own good”; *Cronbach’s α* = 0.81 for T1; *Cronbach’s α* = 0.85 for T2; and *Cronbach’s α* = 0.87 combined T1 and T2), four items measure external regulation (i.e., “because I am supposed to do it.”; *Cronbach’s α* = 0.90 for T1; *Cronbach’s α* = 0.92 for T2; and *Cronbach’s α* = 0.91 combined T1 and T2), and four items measure amotivation (i.e., “there may be good reasons to do this activity, but personally I do not see one”; *Cronbach’s α* = 0.82 combined T1 and T2).

#### 4.4.2. Basic Psychological Need Satisfaction and Frustration

The basic psychological needs satisfaction and frustration scale (BPNSF; B. Chen et al., 2015; 24 items, *Cronbach’s α* = 0.86 for T1 and T2; and *Cronbach’s α* = 0.86 for combining T1 and T2) was used to measure the satisfaction and frustration of basic psychological needs of autonomy, relatedness, and competence. Participants were provided a Likert scale from 1 to 5 (1 corresponds to “not true at all” and 5 corresponds to “completely true”) to assess to what degree each statement is true for him/her at this moment of life. There is a total of 24 items in BPNSF, including four items capturing each needs-satisfaction and frustration, such as autonomy need satisfaction (i.e., “I feel that my decisions reflect what I really want.”; *Cronbach’s α* = 0.80 for T1 and T2; and *Cronbach’s α* = 0.83 combined T1 and T2), autonomy needs frustration (i.e., “I feel pressured to do too many things”; *Cronbach’s α* = 0.85 for T1 and T2; and *Cronbach’s α* = 0.85 combined T1 and T2), relatedness needs satisfaction (i.e., “I feel that the people I care about also care about me”; *Cronbach’s α* = 0.83 for T1; and *Cronbach’s α* = 0.85 combining T1 and T2), relatedness needs frustration (i.e., “I feel excluded from the group I want to belong to”; *Cronbach’s α* = 0.91 for T1 and T2; *Cronbach’s α* = 0.91 combined T1 and T2), competence needs satisfaction (i.e., “I feel confident that I can do things well”; *Cronbach’s α* = 0.81 for T1; *Cronbach’s α* = 0.81 for T2; and *Cronbach’s α* = 0.81 combined T1 and T2), and competence needs frustration (i.e., “I feel disappointed with many of my performances”; *Cronbach’s α* = 0.91 for T1; *Cronbach’s α* = 0.91 for T2; and *Cronbach’s α* = 0.91 combined T1 and T2).

#### 4.4.3. Vitality

The subjective vitality scale (Ryan & Frederick, 1997; 6 items, *Cronbach’s α* = 0.88 for T1; *Cronbach’s α* = 0.91 for T2; and *Cronbach’s α* = 0.94 for combined T1 and T2) was used to measure the vitality experienced by participants. Participants were asked to use a scale of 1 to 7 (1 being “not at all” and 7 being “very true”) to answer the question, “How did you feel during the work episode?” This measurement contained six items (e.g., “I felt so alive I just wanted to burst during that episode”).

#### 4.4.4. General Self-Efficacy

The general self-efficacy scale was used to measure general self-efficacy (G. Chen et al., 2001; 8 items, *Cronbach’s α* = 0.92 for T1; *Cronbach’s α* = 0.93 for T2; and *Cronbach’s α* = 0.92 for combined T1 and T2). First, participants were provided with an explanatory definition of self-efficacy, and then they were asked to use a scale of 1 to 5 (where 1 is “not true at all” and 5 is “completely true”) to assess their general efficacy. There were eight items (e.g., “I am confident that I can perform effectively on many different tasks.”) in this measurement.

### 4.5. Study Two—Measurement Invariance and Statistics Analytic Strategies

We used the means and covariance structure (MACS) analysis as the mechanism and simultaneously tested individual and group mean differences. Multi-group confirmatory factor analysis (CFA) was initially used to test measurement invariance; subsequently, the group means differences in latent factors (experimental and control conditions) were examined with satisfactory scalar invariance model results (Ployhart & Oswald, 2004). In addition, structural equation modeling (SEM) was employed to test the proposed path model in the research model (see Figure 2) using a total experience sample. CFA and SEM analyses were performed using Mplus 8.7 (Muthén & Muthen, 2017), MONOVA, and the descriptive statistical analyses in IBM SPSS Statistics 27 (Armonk, NY, USA).

We reported the fit indices most included in studies from the organizational literature (Medsker et al., 1994): the root mean square error of approximation (RMSEA), the comparative fit index (CFI), and the chi-square statistic. In addition, we considered rough benchmark values for RMSEA as ≤0.05 indicates a good fit, 0.05 to 0.08 indicates a moderate fit, and >0.10 indicates a poor fit (Browe & Cudeck, 1993); and values of CFI ≥ 0.90 to 0.95 indicate an acceptable fit, with values closer to 1.00 indicating a good fit (Hu & Bentler, 1999).

## 5. Study Two—Results

### 5.1. Study Two—Preliminary Analysis

Table 3 contains means and standard deviations for all the self-report variables and the correlations among these variables captured before (T1) and after (T2) the experimental manipulations. As a result, we concluded that our data show no gender bias based on the satisfactory scalar measurement invariance (χ^2^ = 2818.01; CFI = 0.94; TLI = 0.93; RMSEA = 0.05; and SRMR = 0.05) results across all variables between the gender groups. Furthermore, all correlations in Table 3 were in reasonable directions. We also noticed that controlled motivation negatively correlates with relatedness (*r* = −0.11 at T1 and T2; *p* < 0.001) and competence (*r* = −0.09, n.s. at T1; *r* = −0.28 at T2; *p* < 0.001) needs satisfaction as well as positively correlates with autonomy (*r* = 0.67 at T1 and T2; *p* < 0.001), relatedness (*r* = 0.74 at T1; *r* = 0.72 at T2; *p* < 0.001), and competence (*r* = 0.63 at T1; *r* = 0.62 at T2; and *p* < 0.001) needs frustration.

### 5.2. Measurements Invariance, Manipulation Checks, and Group Latent Means Comparisons

First, we conducted a measurement invariance test on the latent factors (autonomy need satisfaction, autonomy need frustration, relatedness need satisfaction, relatedness need frustration, competence need satisfaction, and competence needs frustration) in BPNSF (B. Chen et al., 2015) across the experimental and control conditions to further test the effectiveness of the experimental manipulations (3 × 2 × 1 factorial design). Muti-group CFA confirmed acceptable metric (χ^2^ = 1069.55; df = 547; CFI = 0.95; TLI = 0.91; RMSEA = 0.09; and SRMR = 0.09) invariance, and scalar (χ^2^ = 1198.55; df = 625; CFI = 0.95; TLI = 0.90; RMSEA = 0.09; and SRMR = 0.09) invariance tests.

We compared the latent mean of the factors in BPNSF (i.e., autonomy needs satisfaction/frustration, relatedness needs satisfaction/frustration, and competence needs satisfaction/frustration) across the experimental and control conditions to assess the effectiveness of the experimental manipulations (see Table 4). The results showed that competence needs satisfaction, and the three needs frustration conditions were significantly different from the control group’s latent means. These results confirm the experimental manipulation’s partial effectiveness, mainly in the needs-thwarting experimental conditions. Furthermore, the online word games and surveys were expected to be completed within 10–20 min, a typical “controlling” condition according to the MTurk participant recruiting message; especially because they were instructed that they would be timed for these challenging tasks. Hence, positively encouraging and supportive instructions during the word game could be perceived as not helping the participants’ autonomous self-regulation, but rather as releasing or easing the “controlling” task situation.

**Table 4:** Latent group means comparison results on experimental manipulations (BPNSF) and dependent variables (situational motivation, vitality, and general efficacy).^2^

	**BPNSF** **Latent Means**	**Autonomy Need** **Satisfaction**			**Relatedness Need** **Satisfaction**			**Competence Need** **Satisfaction**			**Autonomy Need** **Frustration**			**Relatedness Need** **Frustration**			**Competence Need** **Frustration**		
**Condition Groups**		**Estimate**	**S.E.**	***p***	**Estimate**	**S.E.**	***p***	**Estimate**	**S.E.**	***p***	**Estimate**	**S.E.**	***p***	**Estimate**	**S.E.**	***p***	**Estimate**	**S.E.**	***p***
1	Control	-	-	-	-	-	-	-	-	-	-	-	-	-	-	-	-	-	-
2	Autonomy needs supportive	−0.17	0.18	0.35	−0.06	0.19	0.75	−0.07	0.17	0.67	0.03	0.17	0.85	−0.02	0.15	0.90	−0.13	0.17	0.44
3	Relatedness needs supportive	0.01	0.16	0.96	0.004	0.19	0.99	0.00	0.16	0.99	−0.19	0.17	0.25	−0.07	0.14	0.62	−0.33	0.16	0.06
4	Competence needs supportive	0.34	0.16	0.03	0.09	0.20	0.83	0.39	0.16	0.01	−0.21	0.17	0.22	−0.35	0.16	0.03	−0.57	0.17	0.001
5	Autonomy needs thwarting	0.001	0.17	0.99	0.07	0.21	0.75	0.11	0.16	0.50	−0.36	0.18	0.04	−0.37	0.16	0.02	−0.50	0.18	0.005
6	Relatedness needs thwarting	0.29	0.16	0.07	0.16	0.18	0.25	0.14	0.16	0.32	−0.24	0.18	0.18	−0.39	0.15	0.01	−0.66	0.17	0.000
7	Competence needs thwarting	−0.40	0.17	0.02	0.20	0.19	0.29	0.19	0.16	0.22	0.17	0.17	0.34	−0.01	0.15	0.96	−0.42	0.17	0.05
	**Latent Group Means**		**Autonomous** **Motivation**					**Controlled** **Motivation**					**Vitality**				**General Efficacy**		
			**Estimate**		**S.E.**		***p***	**Estimate**		**S.E.**	***p***		**Estimate**		**S.E.**	***p***	**Estimate**	**S.E.**	***p***
1	Control		-		-		-	-		-	-		-		-	-	-	-	-
2	Autonomy needs supportive		−0.21		0.14		0.13	−0.33		0.14	0.02		−0.21		0.14	0.13	−0.22	0.15	0.13
3	Relatedness needs supportive		0.07		0.14		0.62	−0.36		0.13	0.02		0.03		0.14	0.83	−0.12	0.14	0.38
4	Competence needs supportive		0.29		0.13		0.03	−0.29		0.13	0.03		−0.01		0.14	0.96	0.23	0.14	0.11
5	Autonomy needs thwarting		−0.15		0.14		0.27	−0.28		0.14	0.05		−0.06		0.13	0.22	0.07	0.14	0.58
6	Relatedness needs thwarting		−0.18		0.12		0.14	−0.30		0.13	0.02		0.04		0.13	0.79	0.18	0.13	0.19
7	Competence needs thwarting		−0.13		0.15		0.37	−0.29		0.14	0.04		−0.08		0.15	0.58	−0.03	0.16	0.85
Post-hoc multiple paired comparison results from MONOVA in dependent variables across the six experimental conditions.																			
**Condition Groups**			**Autonomous Motivation**					**Controlled Motivation**					**Vitality**				**General Efficacy**		
			**Mean Diff.^3^**		**S.E.**		***p***	**Mean Diff.**		**S.E.**	***p***		**Mean Diff.**		**S.E.**	***p***	**Mean Diff.**	**S.E.**	***p***
1	Autonomy needs supportive		0.31		0.20		0.14	−0.53		0.25	0.04		−0.03		0.13	0.30	0.18	0.11	0.10
2	Relatedness needs supportive		0.07		0.20		0.70	−0.42		0.25	0.08		0.06		0.13	0.63	0.11	0.11	0.31
3	Competence needs supportive		0.37		0.20		0.07	−0.67		0.25	0.01		−0.10		0.13	0.44	0.17	0.11	0.14
4	Autonomy needs thwarting		−0.17		0.21		0.41	−0.62		0.25	0.01		−0.32		0.13	0.02	0.04	0.11	0.72
5	Relatedness needs thwarting		−0.20		0.21		0.32	−0.68		0.24	0.01		−0.05		0.13	0.72	−0.05	0.23	0.24
6	Competence needs thwarting		−0.18		0.20		0.38	−0.49		0.25	0.05		−0.10		0.13	0.43	−0.02	0.11	0.80

We then tested and obtained satisfactory results for the scalar invariance, often called the strong invariance (Ployhart & Oswald, 2004), as the multi-group CFA model restrains the same intercepts, latent factors’ loading, and structure of all the dependent variables (i.e., situational work motivation, vitality, and general self-efficacy; χ^2^ = 2241.18; df = 1422; CFI = 0.94; TLI = 0.94; and RMSEA = 0.13; SRMR = 0.08) among the experimental and control groups (3 × 2 × 1 factor design). Sequentially, acceptable chi-square difference tests among nested models for scalar, matrix (χ^2^ = 2098.29; df = 1320; CFI = 0.94; TLI = 0.94; RMSEA = 0.07; and SRMR = 0.08), and configural (χ^2^ = 1984.78; df = 1218; CFI = 0.94; TLI = 0.93; RMSEA = 0.08; and SRMR = 0.06) invariance models were also obtained accordingly. The comparison of group latent mean differences was reported in Table 4. Results show that controlled motivation across all experimental conditions was significantly lower than that of the control group. On the other hand, autonomous motivation was reported to be higher than that of the control group, only in the competence-supportive conditions. There are no significant between-group latent mean differences for vitality and general efficacy across all experimental conditions.

### 5.3. Alternative Mean and Covariance Structure Analyses Results

We also conducted a series of MONOVA analyses, as all dependent variables, including vitality, situational autonomous/controlled motivation, and general efficacies, achieved scalar invariance in the multi-group CFA tests. There was no significant two-way interaction effects noticed between time and experimental conditions. Results support the significant time differences (T1 vs. T2), with only small main effects, in vitality (F [1,737] = 4.13, *p* = 0.04, and ŋ^2^ = 3.61) and autonomous motivation (F [1,737] = 4.50, *p* = 0.03, ŋ^2^ = 9.66). No significant time differences were found in general efficacy and controlled motivation. The significant but minor decrease (i.e., minor change of R^2^) in vitality and autonomous motivation from T1 to T2 (within 30 min) could result from physical and psychological fatigue deduced by repeated exposures to online word games.

In addition, the significant main effects of the experimental conditions were found for controlled motivation (F [1,6] = 4.78, *p* = 0.04, and ŋ^2^ = 5.69) when compared with the control group across the six experimental conditions. Post-hoc paired comparison results show that vitality was significantly lower than in the control group when the basic psychological need for autonomy was thwarted. Most interestingly, controlled motivation was significantly lower than the control groups across the six needs-supportive and thwarting conditions. These results are similar to those obtained from the group latent means comparison results of the multi-group CFA tests.

### 5.4. Alternate Path Model Analyses

To test the path model from self-reported needs satisfaction/frustration to vitality and general efficacy via situational autonomous/controlled motivation (see Figure 2), we first tested the measurement models (χ^2^ = 2550.78; df = 783; CFI = 0.95; TLI = 0.92; RMSEA = 0.05; and SRMR = 0.06) with total needs satisfaction and frustration as independent factors in BPNSF, autonomous/controlled motivation, vitality, and general efficacy using the experience sample (N = 748 across T1 and T2) in Mplus 8.7 (Muthén & Muthen, 2017). Then, the SEM test results support the significant indirect path from the basic psychological needs satisfaction to vitality (*β* = 0.35, *p* < 0.001) via autonomous motivation (*β* = 0.67, *p* < 0.001) but not the negative indirect path via controlled motivation (i.e., the upper independent path in Figure 1). In addition, the indirect paths from the basic psychological needs satisfaction *(β* = 0.64, *p* < 0.001) and frustration (*β* = −0.26, *p* < 0.001) to general efficacy via autonomous (*β* = 0.26, *p* < 0.001) and controlled motivation (*β* = 0.13, *p* < 0.05) were found to be significant in this dataset. These supplementary alternate path model results indicate that the path model from basic psychological needs to subjective well-being (i.e., vitality) and self-identified work attitude (i.e., general efficacy) via situational motivation could be altered by manipulating whether the basic psychological needs were supported or thwarted in the situation. However, the specific path may vary in terms of dimensions of fluidity. Though this study focused mainly on the effectiveness of online theory-based manipulations, we are confident that the replication of cross-sectional empirical results of previous empirical evidence could advance the research on temporal patterns in work motivation and its consequences on employees’ self-regulation under SDT (i.e., Wang & Panaccio, 2022; Bidee et al., 2016). Both longitudinal and experimental research can lay the solid theoretical and practical groundwork for advancing future research on theory-based interventions.

## 6. General Discussions

Two independent empirical studies were conducted in this research to answer the research question of how self-determination theory-based interventions change employees’ motivation and motivational consequences within short time frames (i.e., within an hour, within a few weeks or months). Field study one confirmed that the one-day training interventions significantly improved both managers’ and subordinates’ self-reported needs satisfaction and frustration. In addition, subordinates reported managers’ reduced needs-thwarting behavior six weeks after the training workshop. However, training interventions in study one resulted in no significant changes in managers’ and employees’ work motivation (i.e., at the life domain level; Vallerand, 1997). On the other hand, online study two consistently showed a reduction in situational controlled motivation in the planned assignments (i.e., two online word games) across the needs-supportive and thwarting conditions compared to the control group among Amazon Mechanical Turk participants.

The results reveal interesting change patterns through two experimental research designs, encompassing both online and on-ground interventions, within a relatively short timeframe. In summary, theory-based interventions (i.e., one-day on-ground in-person training vs. a few minutes of an online word game) appeared to be an effective means of deconstructing the change mechanisms and temporal patterns in basic psychological needs and the domain (study one), as well as situational (study two) work motivation. Theoretically speaking, although the interventions in this research focused on different conceptual levels (i.e., a few lines of explanation in the instructions to online word games in study two vs. a full-day training workshop in study one; Vallerand, 1997) and temporal intervals (i.e., a few weeks vs. a few minutes/hours; Shipp & Cole, 2015), we successfully deconstructed the manifestations of the changes in situational and domain levels of work motivation as well as basic psychological needs satisfaction/frustration. Evidence from this research helps scholars further map different elements of motivation (i.e., starting and ending levels, speed, and directions of change) within the SDT framework.

In addition, we included and tested changes in work motivation by using different measurements for work motivation, for example, domain-level motivation at work scale (Gagné et al., 2015) for study one and situational motivation scale (Guay et al., 2000) in study two to capture the variation in dynamic patterns in different conceptual levels of motivation interventions (Vallerand, 1997) in this research. We can also see that a one-day training intervention mainly captured the changes in the basic psychological needs satisfaction/frustration compared to a few minutes of an online intervention, affecting situational controlled motivation across the experimental conditions. It is also interesting to see that domain-level work motivation was not significantly changed in study one, while the situational level work motivation, mostly controlled motivation across needs satisfaction/frustration conditions, changed in study two. Hence, future complex research designs are warranted to investigate the dynamic temporal properties (i.e., starting levels, speed and directions of changes) of work motivation and motivational consequences across conceptual levels (i.e., trait, domain, and situational levels) at work.

The concept of intervention originated in developmental psychology when Kanfer and Goldstein (1991) described methods used to intervene as designed to help people change for the better. Psychologists such as Adelman and Taylor (1994) illustrated unintentional and intentional transactions and outcomes using intentional interventions. However, intentional intervention aims to produce intended outcomes through planned processes. According to Adelman and colleague (1994), the intended outcomes encompass maintenance, change, or transformation with respect to problematic or nonproblematic conditions of the target systems. The combined processes of intentional intervention may or may not produce the intended outcomes and may also produce unintended outcomes; furthermore, some outcomes may be adverse. In online study two, the original purpose of the manipulation conditions was to deduce the impact of the momentary changes in needs satisfaction and frustration on situational motivation (autonomous vs. controlled), vitality, and self-efficacy. Results in study two revealed that only situational controlled motivation was significantly and consistently lower across all experimental conditions (i.e., autonomy, relatedness, and competence needs-supportive vs. thwarting conditions). In this case, it was unexpected that situational controlled motivation was reduced across the needs-supportive conditions, where situational autonomous motivation was expected to change.

The theoretical reasoning behind the consistently reduced situational controlled motivation across experimental conditions in study two could be complex. First, needs-supportive conditions may ease the participants’ performance pressure (i.e., being timed) and provide connections and empathy so that their situational controlled motivation (i.e., the extrinsic drive for task completion) was significantly reduced compared to the control group. Second, the needs-thwarting conditions overall reduced the motivation levels via depleting participants’ options, depersonalizing their past effort and performance. Surprisingly, situational autonomous motivation remained unchanged across the three needs-thwarting conditions in this study. Amazon MTurk workers, our research participants of online study two, could be mainly extrinsically regulated to complete minor tasks for minimum financial benefit; hence, the needs-supporting conditions could effectively reduce their main reason, being their extrinsic drive to make quick and brainless money, for completing the tasks and short surveys around 20–30 min. Though both needs-supporting and thwarting conditions resulted in reduced situational controlled motivation, different psychological mechanisms and processes could interact and coexist. Hence, we could also consider the integration of other theories that may explain the reduced situational controlled motivation across experimental conditions. However, which complementary theories can be used to explain the momentary activation or suppression of psychological processes is yet to be established, as we have not captured emotion, affect, or other situational factors in study two.

As a result, this online study two may only effectively pick up all the changes in the situational controlled motivation^4^ compared to other online or experimental sampling methods. Hence, further research may need to investigate the effectiveness of this type and other short online interventions in deducing motivational changes via manipulations of basic psychological needs across different situations and motivational intentions in people. In addition, findings in this intervention study also show the importance of studying both types of autonomous and controlled motivation simultaneously with consideration of the conceptual levels and dynamic processes/consequences of motivation (i.e., Wang & Panaccio, 2022, 2023), along with accumulated empirical studies using SDT interventions mainly focusing on feedback mechanisms (i.e., Gradito Dubord et al., 2022; Jungert et al., 2022), which were mainly similar to field study one. 

The practical implications of our findings are mixed. Practitioners are the ones who bring management science and research evidence to real-world problem-solving situations; hence, both caution and bold trial-and-error experiments are needed by them when applying current findings to field experiment designs. For example, findings in study two about reduced controlled motivation across different experimental conditions can be easily replicated with individual managers’ daily interactions with subordinates. However, it may only be statistically validated through the real-time capture of momentary data (i.e., real-time data captured through wearable/mobile devices), which can provide clear guidance for the cognitive and behavioral awareness of first-line managers through timely corporate training. In addition, practitioners can also incorporate a control group design into their organizational development programs to capture the effect of employees’ learning experiences (i.e., to eliminate the research limitation in our first field study). With the advancement of technology in management (i.e., AI and/or big data in applied algorithmic control), scholars and practitioners can collaborate to enhance the accumulation of empirical evidence and the development of theories in management science.

## 7. Limitations

This research also has limitations. First, field study one did not have a control group due to the small sample size and tight training schedule, which may hinder the external validity of the research findings. Second, study one used two independent scales measuring the generic measurement of basic psychological needs satisfaction and frustration (i.e., BPNSF); participants reported moderate to high needs satisfaction and low needs frustration across experimental and control conditions. Using more sensitive and situational-specific measurements for both needs satisfaction and frustration (i.e., B. Chen et al., 2015) may better validate the momentary experimental manipulations in future research. Third, online study two used Amazon Mechanical Turk participants, which may unintentionally restrict the variance in situational autonomous motivation in this study. Future intervention research could use broader, population-representative participants recruited from corporate employees. Lastly, the potential limitation of not being able to detect significant change in targeted autonomous motivation in online study two could result from the lack of considering other complementary theories (i.e., dual process model of motivation and emotion; Strack & Deutsch, 2004), capturing additional situational psychological constructs, for further modification in theory-based interventions (i.e., online game instructions), rather than focusing on only basic needs satisfaction/frustration. Careful examination and rewording of such interventions may also be needed for future studies.

## 8. Conclusions

It is not difficult to conclude that there has been limited research on self-determination theory-based interventions, as evidenced by a literature review (Day et al., 2022; Guay, 2022) or meta-analytical research (Pandey et al., 2018). Furthermore, most existing intervention research has been conducted in clinical, developmental, educational, and sports psychology; however, there is currently a scarcity of empirical research on theory-based interventions in management. With this in mind, we advocate for more scientific research on theory-based interventions to inform evidence-based management practices for strategic employee development and organizational change programs in the future.

## 9. Declarations

### Compliance with Ethical Standards

The authors obtained from their University’s ethics board the ethical approval for using an online survey (with informed consent) to collect data from human participants

The authors have no relevant financial or non-financial interests to disclose.

The authors have no competing interests to declare that are relevant to the content of this article.

All authors certify that they have no affiliations with or involvement in any organization or entity with any financial interest or non-financial interest in the subject matter or materials discussed in this manuscript.

The authors have no financial or proprietary interests in any material discussed in this article.

## Figures and Tables

**Table 1:** Means, standard deviations, and correlations for the self-report variables (T1 and T2) in the field for study one.

Managers	T1 Mean	T1 S.D.	1	2	3	4	5	6	7	8	9	10	T2 Mean	T2 S.D.
1. Autonomous motivation	5.74	0.75	-	−0.12	0.73 **	0.16	0.52	0.40	0.04	−0.44	−0.48	−0.50	5.75	0.78
2. Controlled motivation	2.38	1.13	−0.43 **	-	−0.46	0.33	−0.11	0.35	−0.05	0.21	−0.44	−0.42	2.60	0.72
3. Needs-supportive behavior	5.75	0.54	0.53 **	−0.37 *	-	−0.15	0.30	0.24	−0.03	−0.18	0.03	−0.10	5.79	0.58
4. Needs-thwarting behavior	1.36	0.62	−0.29	0.52 **	−0.38 *	-	0.57	0.04	−0.15	0.16	−0.18	−0.21	1.07	0.16
5. Autonomy needs satisfaction	5.57	0.85	0.24	−0.33	0.14	−0.44 *	-	0.51	0.52	−0.52	0.16	−0.59 *	5.79	0.86
6. Relatedness needs satisfaction	5.94	0.78	0.03	0.14	0.17	−0.18	0.46 *	-	0.05	−0.17	0.06	−0.75 **	5.92	0.85
7. Competence needs satisfaction	6.13	0.51	0.50 **	−0.52 **	0.24	−0.27	0.48 **	0.11	-	−0.12	0.05	0.05	6.19	0.63
8. Autonomy needs frustration	3.18	1.13	0.03	0.25	0.32	0.17	−0.63 **	−0.15	−0.31	-	−0.17	0.51	2.81	1.19
9. Relatedness needs frustration	1.70	0.70	−0.06	0.06	−0.03	0.09	−0.54 **	−0.56 **	−0.17	0.43 **	-	0.86 **	1.34	0.57
10. Competence needs frustration	1.65	0.76	−0.11	0.23	0.01	0.29	−0.59 **	−0.41 *	−0.27	0.54 **	0.75 **	-	1.38	0.58
**Direct Subordinates**														
1. Autonomous motivation	5.54	0.84	-	−0.05	0.32 *	−0.22	0.23	0.18	0.42 **	−0.14	−0.17	−0.17	5.56	0.77
2. Controlled motivation	2.43	0.93	−0.14	-	−0.23	0.21	−0.26	−0.14	−0.04	0.33 *	0.05	0.27 *	2.32	1.01
3. Needs-supportive behavior	5.55	0.96	0.31 **	−0.26 **	-	−0.66 **	0.50 **	0.31 *	0.37 **	−0.05 **	−0.29 *	−0.56 **	5.65	1.09
4. Needs-thwarting behavior	1.56	1.09	−0.13	0.23 **	−0.64 **	-	−0.47 **	−0.21	−0.35 **	0.45 **	0.42 **	0.58 **	1.41	0.78
5. Autonomy needs satisfaction	5.40	0.95	0.32 **	−0.32 **	0.41 **	−0.32 **	-	0.58 **	0.43 **	−0.79 **	−0.60 **	−0.78 **	5.63	0.91
6. Relatedness needs satisfaction	5.97	0.69	0.31 **	−0.11	0.27 **	−0.16	0.49 **	-	0.28 *	−0.47 **	−0.68 **	−0.58 **	5.72	1.03
7. Competence needs satisfaction	5.73	1.01	0.32 **	−0.05	0.30 **	−0.28 **	0.32 **	0.31 **	-	−0.29 *	−0.16	−0.29 *	5.76	0.77
8. Autonomy needs frustration	3.00	1.30	−0.19 *	0.32 **	−0.42 **	0.29 **	−0.75 **	−0.36 **	−0.18 *	-	0.54 **	0.82 **	2.41	1.30
9. Relatedness needs frustration	1.97	1.08	−0.24 **	0.15	−0.28 **	0.33 **	−0.54 **	−0.60 **	−0.25 **	0.43 **	-	0.76 **	1.84	1.22
10. Competence needs frustration	1.64	0.86	−0.16	0.27	−0.48 **	0.50 **	−0.68 **	−0.53 **	−0.32 **	0.65 **	0.65 **	-	1.56	0.86

Note: managers *n* = 22 for T1 and T2; subordinates *n*’ = 61 for T1 and T2; S.D. = standard deviation; autonomous motivation includes both intrinsic motivation and identified regulation; controlled motivation includes extrinsic regulation and introjected regulation; * *p* < 0.05; and ** *p* < 0.001.

**Table 2:** Univariate ANOVA test results (managers and subordinates’ self-report results across T1 and T2) in field study one.

Dependent Variables		Mean Square	DF	F	*p* Level	ŋ^2^
**Managers**						
Autonomous motivation	Contrast	0.002	1	0.006	0.938	0.000
	Error	0.407	43			
Controlled motivation	Contrast	3.043	1	2.487	0.127	0.090
	Error	1.224	43			
Needs-supportive behavior	Contrast	0.053	1	0.182	0.673	0.007
	Error	0.292	43			
Needs-thwarting behavior	Contrast	0.978	1	3.203	0.086	0.114
	Error	0.305	43			
Basic psychological needs satisfaction	Contrast	4.748	1	23.495	0.000	0.484
	Error	0.202	43			
Basic psychological needs frustration	Contrast	0.521	1	7.462	0.001	0.23
	Error	0.070	43			
**Direct Subordinates**						
Autonomous motivation	Contrast	0.333	1	0.536	0.47	0.004
	Error	0.621	121			
Controlled motivation	Contrast	0.233	1	0.279	0.60	0.002
	Error	0.835	121			
Managers’ needs satisfaction behavior	Contrast	0.997	1	1.225	0.27	0.01
	Error	0.814	121			
Managers’ needs-thwarting behavior	Contrast	2.549	1	5.088	0.03	0.09
	Error	0.501	121			
Autonomous needs satisfaction	Contrast	6.58	1	8.329	0.005	0.06
	Error	0.79	121			
Relatedness needs satisfaction	Contrast	66.14	1	141.11	0.000	0.52
	Error	0.469	121			
Competence needs satisfaction	Contrast	2.766	1	5.991	0.016	0.044
	Error	0.462	121			
Basic psychological needs satisfaction	Contrast	3.466	1	28.22	0.000	0.189
	Error	0.123	121			
Autonomous needs frustration	Contrast	28.971	1	19.619	0.000	0.131
	Error	1.477	121			
Relatedness needs frustration	Contrast	1.532	1	2.267	0.135	0.017
	Error	0.0676	121			
Competence needs frustration	Contrast	3.468	1	3.346	0.07	0.025
	Error	1.036	121			
Basic psychological needs frustration	Contrast	1.463	1	5.047	0.03	0.094
	Error	0.290	121			

Note: tests were based on the linearly independent pairwise comparisons among the estimated marginal means.

**Table 3:** Means, standard deviations, and correlations for the self-report variables (T1 and T2) in the online study two.

	T1 Mean	T2 S.D.	1	2	3	4	5	6	7	8	9	10	11	12	T2 Mean	T2 S.D.
1. Age	36.38	11.03	-	0.51 **	−0.05	−0.08	0.08	−0.04	0.13 *	−0.15 **	0.04	−0.12 *	0.00	0.07	36.38	11.03
2. Organizational tenure	9.44	7.64	0.51 **	-	0.14 **	0.10	0.10	0.12 *	0.05	0.15 **	0.12 *	0.13 *	0.12 *	0.04	9.44	7.64
3. Autonomous motivation	4.79	1.38	0.03	0.11 *	-	0.54 **	0.54 **	0.23 **	0.35 **	0.47 **	0.61 **	0.24 **	0.75 **	0.45 **	4.50	1.55
4. Controlled motivation	4.05	1.80	−0.13 *	0.06	0.58 **	-	0.03	0.67 **	−0.11 *	0.72 **	−0.28 **	0.62 **	0.34 **	0.04	4.06	1.79
5. Autonomy needs satisfaction	3.95	0.72	0.10	0.07	0.48 **	0.02	-	−0.21 **	0.69 **	0.02	0.72 **	−0.23 **	0.62 **	0.70 **	3.82	0.81
6. Autonomy needs frustration	3.27	1.08	−0.04	0.12 *	0.27 **	0.67 **	−0.21 **	-	−0.22 **	0.74 **	0.08	0.76 **	0.05	−0.22 **	3.25	1.10
7. Relatedness needs satisfaction	4.06	0.82	0.11 *	−0.05	0.29 **	−0.11 **	0.74 **	−0.21 **	-	−0.26 **	0.49 **	−0.26 **	0.42 **	0.55 **	3.94	0.86
8. Relatedness needs frustration	2.93	1.28	−0.06	0.17 **	0.41 **	0.72 **	−0.09	0.74 **	−0.31 **	-	0.29 **	0.76 **	−0.31 **	−0.13 *	2.97	1.28
9. Competence needs satisfaction	4.06	0.071	0.17 *	0.04	0.33 **	−0.09	0.74 **	−0.25 **	0.70 **	−0.23 **	-	0.05	0.61 **	0.62 **	3.72	0.79
10. Competence needs frustration	2.88	1.26	−0.09	0.17 **	0.20 **	0.63 **	−0.23 **	0.76 **	−0.31 **	0.81 **	−0.40 **	-	0.06	−0.36 **	2.98	1.23
11. Vitality	3.62	0.89	0.01	0.09	0.70 **	0.35 **	0.61 **	0.04	0.44 **	−0.22 **	0.50 **	0.04	-	0.56 **	3.43	0.998
12. General efficacy	3.91	0.78	0.09	0.04	0.53 **	0.06	0.70 **	−0.24 **	0.57 **	−0.16 **	0.75 **	−0.36 **	0.64 **	-	3.80	0.84

Note: *n* = 362 for T1; *n* = 360 for T2; S.D. = standard deviation; * *p* < 0.05; and ** *p* < 0.001.

**Figure A1:**
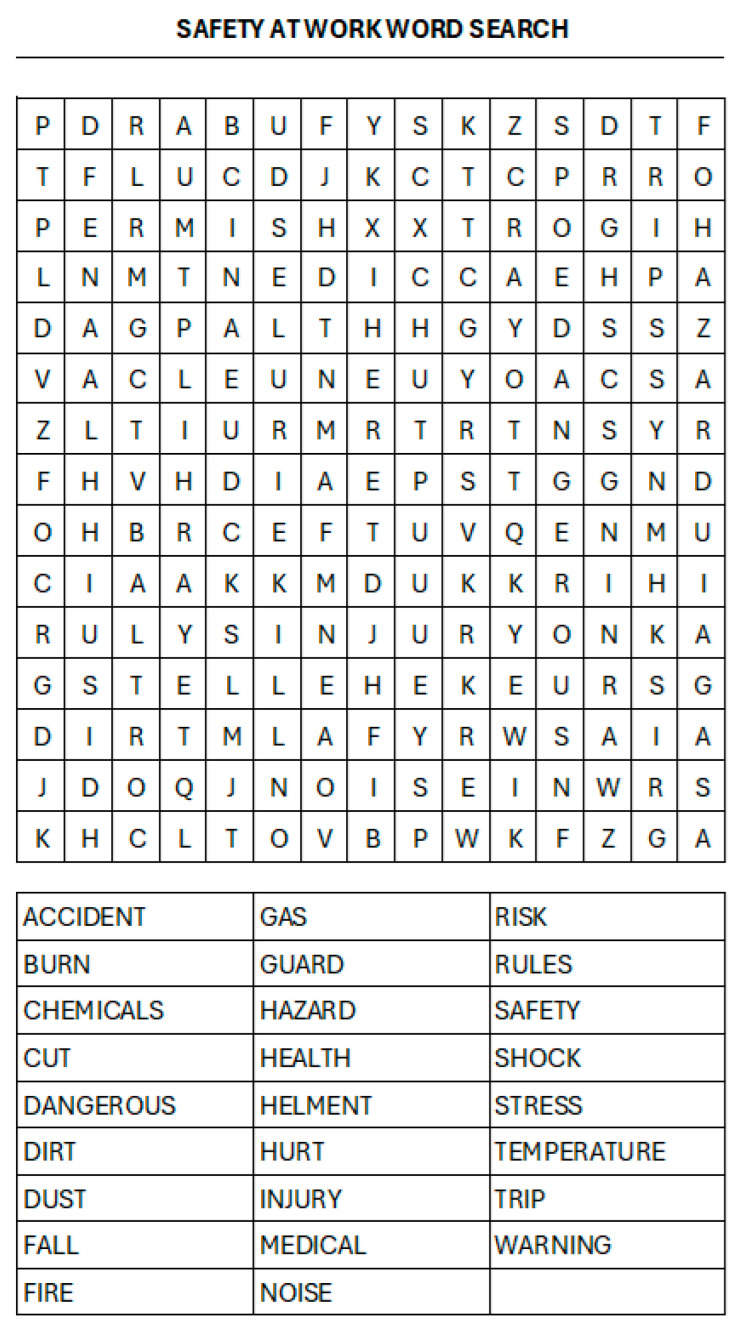
The first word search task in the experiment (Study Two).

**Figure A2:**
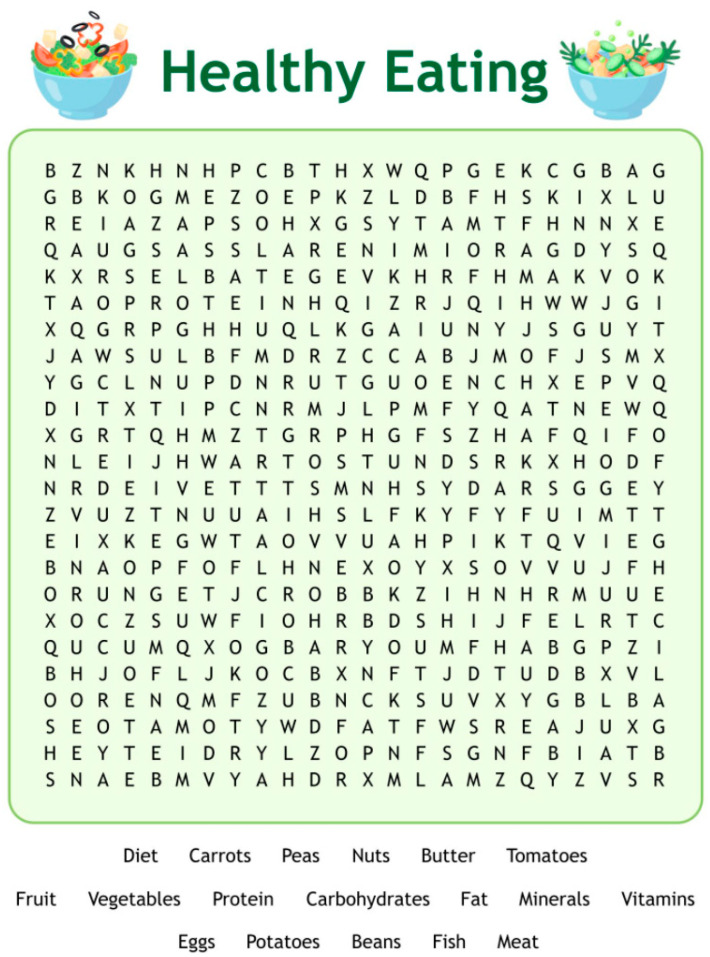
The second word search task in the experiment (Study Two).

## Data Availability

The datasets generated during and/or analyzed during the current study are available from the corresponding author on reasonable request.

## Notes

1 The seventh condition within the 3 × 2 × 1 factorial design is the control condition without any experimental manipulation of situational work motivation and basic psychological needs.

2 Latent means in group condition 1 (control group) were set to be zero when compared with other group conditions.

3 Mean difference = Mean of condition group − Mean of the control group.

4 Online study two used situational work motivation scale (Guay et al., 2000), which only measures and captures extrinsic situational regulation as the situational controlled motivation.

## Appendix A

You are chosen to do a few quick cognitive exercises to be selected for the next managerial training program (the required employees’ developmental program for the candidates to be promoted to supervising/managerial positions) in your organization. It is a simple task that requires both accuracy and efficiency. Please read the instructions for the exercise carefully.

**Table A1:** Instruction statements (six experimental conditions) for the practice run.^5^

	Autonomy	Relatedness	Competence
**Supportive Condition**	**I**	**II**	**III**
	In this exercise, we just want you to play around with it, learning to do it your own way. You can choose which one to do, and you can choose how you want to try it first. Just try to get into it and see where it goes.	Just to remind you: remember, we care about you and your individual learning style. So, please be sure to remember what you were thinking and feeling, so we can ask for your reactions later. Just remember that we know you are a unique individual with your own learning style. We are focused on trying to understand you personally, not just the problem itself.	One thing to keep in mind is that this task is quite challenging. The task involves finding as many words as possible, and beginners find that they only scratch the surface of the possible words. Just do the best you can, and you will improve quickly. I have confidence in you!
**Thwarting condition**	**IV**	**V**	**VI**
	In this exercise, you must do it right, learning to do it before the time out. In order to achieve experimental control, we cannot let you have any choice but to do the one you are assigned first, or about the level of difficulty. We know what this exercise is about, so just follow the instructions exactly, please.	Just to remind you: remember, we are not really interested in your reactions and individual learning style. So, please keep your questions and observations to yourself during the exercise. You may be thinking that you do not like it, but that does not matter. Remember, to us, you are just one anonymous participant, the same as everybody else. We are focused on trying to understand the effectiveness of the assessment, not you personally.	One thing to keep in mind is that this task is quite difficult. The task involves finding as many words as possible, and beginners (such as you) usually do not find very many words. Still, do it as you are instructed to do, even if it seems hard. Maybe you will be lucky!

Then, participants will be shown the first Word Search Task^6^ and be instructed that he/she will be given 3 min to look at the word puzzle to find as many as possible words in it.

“You need to find as many words as you can in this grid. Write each one down, as you see it, as many as you can in the 3 min.”

After the time for the first exercise is up, the following statement would be shown to proceed to the second round of exercise.

**Table A2:** Instruction statements (six experimental conditions).^7^

	Autonomy	Relatedness	Competence
**Supportive Condition**	**I**	**II**	**III**
	Now that you have a basic understanding of the exercise, you now have a choice of your final exercise based on the level of difficulty.	It is time for the final timed exercise. We can sympathize with what you might be feeling now: you are not sure you like tests such as this. Just remember that we know you are a unique individual with your own learning styles. We are focused on trying to understand you personally, not just the exercise itself.	The first challenge is to give you a sense of how well you can do at the beginning. In fact, people often do quite well at this, initially. Just relax and get into it; we have confidence that you will do well.
**Thwarting condition**	**IV**	**V**	**VI**
	Now that you have a basic understanding of the task, we cannot offer you any choice of the level of difficulty in these word tables. Now you need to proceed to the next exercise immediately.	It is time for the final timed exercise. You may be thinking that you do not like tests, but that does not matter. Remember, to us, you are just one anonymous participant, the same as everybody else. We are focused on trying to understand the validity of the exercise, not you personally.	The first challenge is to give you a sense of how poorly you do at the beginning. In fact, people often do quite badly at this initially. Just try as hard as you can, and hopefully, you will not do too badly in the final task.

**“You need to find as many words as you can in this grid.** Write each one down, as you see it, as many as you can in the 3 min.”
